# Regions of interest multivariate curve resolution for liquid chromatography with ion mobility high-resolution tandem mass spectrometry: analysis of plastic additives

**DOI:** 10.1007/s00216-026-06632-w

**Published:** 2026-06-22

**Authors:** Ana Torres-Agullo, Roma Tauler, Silvia Lacorte, Tytus D. Mak, Yamil Simón-Manso

**Affiliations:** 1 Institute of Environmental Assessment and Water Research of the Spanish Research Council (IDAEA-CSIC), Jordi Girona 18-26, 08034 Barcelona, Spain; 2 Department of Chemical Engineering and Analytical Chemistry, Universitat de Barcelona, Diagonal 645, 08028 Barcelona, Spain; 3 Mass Spectrometry Data Center, Biomolecular Measurement Division, National Institute of Standards and Technology (NIST), Gaithersburg, MD 20899 USA

**Keywords:** ROIMCR, Non-targeted analysis, Ion mobility mass spectrometry, Microplastics, Collision cross-section

## Abstract

Ion mobility (IM) enhances liquid chromatography-mass spectrometry identification capabilities by providing an additional dimension that improves peak capacity, mass detection, and sensitivity, being particularly valuable for untargeted analysis. However, the high dimensionality and large amount of information in the datasets obtained in this type of analysis are a big challenge for data processing, slowing its broader application and potential. The regions of interest multivariate curve resolution (ROIMCR) is a chemometrics approach based on the bilinear model intrinsic structure of the data. It represents an effective strategy for efficient feature extraction, deconvolution, and resolution of overlapping signals in complex datasets without losing relevant information and maintaining instrumental mass accuracy. In this work, the application of ROIMCR is shown for the first time for liquid chromatography ion mobility mass spectrometry (LC-IM-MS) in data-dependent acquisition (DDA) mode, including MS1 and MS2 datasets for the analysis of plastic additives. An improvement in the discrimination of isomeric species and minimization of interferences was shown by working with mobility profiles. IM-MS data from five microsphere standards and three polyethylene (PE) plastic leachates, containing complex mixtures of unknown additives, were analyzed and resolved, and collision cross-section (CCS) values were calculated for each component. Using ROIMCR, 14 different plastic additives, including phthalates, siloxanes, and phosphates, present in the original PE polymers, were identified. Our findings highlight that ROIMCR provides an effective and scalable tool for resolving complex IM datasets and enhancing the identification of potential additives associated with microplastics.

**Supplementary Information:**

The online version contains supplementary material available at https://doi.org/10.1007/s00216-026-06632-w.

## Introduction

Ion mobility instruments have been used since the 1970 s in military and security contexts, mainly as explosives and drug detectors [1]. IMS principle relies on the separation of ions in an inert gas under the effect of an electric field; that is, the separation is based on the ions’ different velocities [2]. In recent years, IMS has dramatically increased the scientific community’s attention due to the latest advances and new applications. Specifically, combining ion mobility with mass spectrometry (IM-MS) has revolutionized the field of mass spectrometry by providing value-added data with a new dimension of molecular separation [3].

LC-IM-MS three-dimensional (3D) analysis has strongly increased sensitivity, selectivity, peak capacity, and chemical noise reduction [4]. The ion mobility dimension added to LC-MS provides complementary separation information that allows the discrimination of isobaric and isomeric species, including regioisomers, geometric isomers, or chiral stereoisomers [5]. When coelutions appear in liquid chromatography (LC), the different spatial conformation of the molecules leads to different mobilities (K) in the gas phase and thus to their separation in the IM dimension on a millisecond timescale [6]. Measured mobility is a function of experimental conditions (e.g., temperature and pressure). Thus, a reproducible property under defined experimental conditions, such as collision cross-section (CCS), is commonly used as a reliable indicator for compound identification [2, 7].

The incorporation of ion mobility as an additional analysis dimension, combined with the large datasets generated, implies a substantial challenge for data handling and interpretation. As a result, many studies rely mainly on the CCS value as a complementary parameter for untargeted identification, rather than fully exploiting the available multidimensional information. Advanced multivariate data analysis approaches are required to integrate IM information with MS fragmentation data and resolve the multiple chemical species present in complex mixtures. In this framework, the regions of interest multivariate curve resolution (ROIMCR) method represents an effective strategy for resolving overlapping signals in the analysis of large MS datasets without losing relevant information or compromising instrumental mass accuracy [8–10]. Initially, the ROI approach compresses and filters the large raw datasets while maintaining the accuracy of the original measurements. Second, the multivariate curve resolution alternating least squares (MCR-ALS) method allows the analysis of multicomponent systems with strongly overlapping signals of multiple analytes present in the samples (i.e., IM-MS) by performing a bilinear factor decomposition of the previously compressed regions of interest (ROI) data, resolving the elution/mobility and spectra profiles of the sample constituents [8, 11, 12]. ROIMCR has already been successfully applied in previous research studies, including, among others, the non-targeted analysis and identification of perfluoroalkyl substances (PFAS) in biological matrices using LC with high-resolution mass spectrometry (HRMS) [13], of extracts from passive samplers deployed in a wastewater treatment facilities [14], of persistent organic pollutants (POPs) in dust with gas chromatography (GC) coupled to HRMS [15], or even of pharmaceutical compounds in two-dimensional liquid chromatography (LCxLC) datasets [16]. Therefore, rather than reducing ion mobility information to a single CCS value, ROIMCR exploits full ion mobility profiles as an additional structured new dimension, enabling improved component resolution, interference minimization, and chemically meaningful analysis of mixtures.

In the present study, ROIMCR is proposed for the first time for modeling LC-IM-MS data in the analysis, resolution, and identification of additives in PE microplastics (MP) leaching tests. MP and associated plastic additives (e.g., plasticizers, flame retardants, heat and light stabilizers, or antistatic agents) used during polymer manufacture are a global pollution issue due to their ubiquitous presence in the environment [17, 18]. Hence, understanding the leachability of additives and polymer composition is essential for the environmental risk assessments of MP [19]. Therefore, in this study, PE microsphere standards and real PE plastic fragments, representing complex mixture samples containing unknown additives, were analyzed by LC-IM-MS, and the data matrices generated were processed by ROIMCR, focusing on the multidimensional structure of these datasets and on the optimal strategies for data analysis. Given the lack of prior knowledge about their chemical composition, a non-targeted analysis approach was adopted to resolve and characterize the leachate components.

## Experimental section

### Chemicals and materials

PE engineered microspheres (1 g/cm^3^, size ranges between 45–53, 106–125, 300–355, 500–600, and 850–1000 µm) (Cospheric, Santa Barbara, USA) were purchased as commercial standards. HPLC-grade water (Honeywell, Charlotte, NC, USA), methanol, and isopropanol supplied by Supelco (Bellefonte, PA, USA) were used. Moreover, PE raw polymer was obtained from Polymer Sample Kit #205 (Scientific Polymer Products Inc., USA). As stated by the manufacturer, this kit contains 100 individual different polymers for reference samples, analytical studies, and small-scale evaluations.

### Sample preparation and leaching experiments

A Bead Ruptor Elite (OMNI International, USA) was used to pulverize the polymer (Polymer Sample Kit #205) in 2-mL reinforced tubes filled with 2.8-mm ceramic beads to provide an efficient grinding. PE was milled in a single step in operating conditions of 250 rpm during 10 s, to a mean diameter of 14.8 µm. A smaller polymer mean size (13.1 µm, diameter) was obtained by repeating the grinding cycle five times. Finally, the smallest mean PE mean size (11.7 µm, diameter) was obtained by grinding during 15 cycles. To prevent heat generation and polymer melting, a dwell time of 1 min was set between cycles, and there was an additional rest period (5 min) after every five cycles. Particle size distributions were determined by flow imaging microscopy using a FlowCam system (Fluid Imaging Technologies, Inc., Yarmouth, Maine, USA), equipped with a high-resolution digital camera, allowing measurement of particle diameters based on image analysis in real time. After the grinding, microplastic particles were placed and stored in glass vials. During the process, an antistatic gun (Milty Zerostat 3, Sigma-Aldrich, USA) was used to avoid MP surface attachment. Table S1 includes the sample code details.

Generated MP (5 mg) were immersed in isopropanol (2 mL) and placed in 15-mL Pyrex glass test tubes capped with screw caps to avoid solvent evaporation. The ample headspace in the vials helped to prevent overpressure. Tubes were shaken under constant conditions (300 rpm, 50 °C) for 24 h using an Eppendorf ThermoMixer® C (Eppendorf, Hamburg, Germany). The mixture was centrifuged (GLC-4 Benchtop Centrifuge, Sorvall Instruments, Thermo Fisher Scientific, MA, USA) at 3000 rpm for 10 min to precipitate microplastic particles. Finally, the leachate was filtered using PTFE syringe filters (0.45 µm pore size, 25 mm diameter, HPTFE, Whatman, Kent, UK) and stored in glass vials. Three independent replicates (*n* = 3) were prepared for each PE type and particle size. All sample preparation was performed using glass material to minimize possible plastic-derived contamination. Procedural isopropanol blanks were also included (*n* = 5), prepared, and processed under the same experimental conditions as the samples, including shaking, centrifugation, and filtration.

### LC-IM-MS analysis

Leachate solutions were analyzed by ultra-high performance liquid chromatography (UHPLC, Thermo Scientific Vanquish Flex, Thermo Scientific, MA, USA) coupled to a timsTOF Pro 2 (Bruker, Germany). A reversed-phase column, CHS C18 2.1 × 100 mm with a particle size of 1.7 µm, was used for the chromatography separation. A 15-min gradient was used with mobile phase A (water:formic acid 99.9:0.1%) and mobile phase B (methanol:formic acid 99.9:0.1%) with the following program: time, %B; 0, 1; 1, 1; 9, 80; 12, 99; 13, 99; 14, 1; 15, 1}. The instrument was equipped with an integrated atmospheric pressure chemical ionization (APCI) probe, and acquisition was done at a mass range from 30 to 1000 *m/z*. The column compartment temperature was set at 35 °C. The ion mobility range (1/*K*_0_) was scanned from 0.1 to 1.5 V s cm^−2^. The instrument was operated in DDA and parallel accumulation serial fragmentation (PASEF) modes, with a ramp rate of 9.42 Hz and two ramps, resulting in a cycle time of 0.32 s. A fixed-intermediate collision energy of 18 eV and an exclusion time of 18 s were used. The instrument was operated in high-resolution mode, providing a resolving power of 50,000 (FWHM). Details about source, tune, and TIMS parameters are included in the supplementary information (Table S2). The vendor-recommended four-point linear model was used for calibration. This calibration typically maps three ions with known 1/*K*_0_ values from a standard (e.g., *m*/*z* 622, 922, and 1222 in positive mode) to their measured elution voltages. A linear regression model was built using the measured mobilities of the calibration ions from the tune mix. The supplementary information (Table S3) provides details on the tune mix used as calibration reference, as well as the linear regression parameters. Quality control and assurance measures were implemented throughout the leaching experiments to ensure data reliability. Both instrumental (*n* = 10) and procedural blanks (*n* = 5) were also analyzed to identify potential instrumental background and cross-contamination during sample preparation and data processing.

In total, three distinct datasets were generated from the leaching solutions for analysis: (a) dataset 1 is a single PE microsphere standard leachate (size fraction 45–53 µm, S1) which was initially processed to compare LC-MS and LC-IM-MS performance; (b) dataset 2 corresponds to five commercially available PE microspheres leachates (from the same standard, with different size ranges: 45–53 (S1), 106–125 (S2), 300–355 (S3), 500–600 (S4), and 850–1000 µm (S5)); and (c) dataset 3 is the home-ground PE polymer leachate samples from the Polymer Sample Kit #205, processed to obtain three different particle sizes (14.8 (K1), 13.1 (K2), and 11.7 µm (K3)).

### Data analysis

#### ROIMCR analysis

Details about ROI selection and MCR-ALS application in the ROIMCR analysis of the microplastic samples are given in the Supplementary Material (Text S1) and in previous works [8–16, 20, 23]. The ROI procedure was selected as a preprocessing and compression methodology to handle the large and complex datasets generated by LC-IM-MS. MCR-ALS is used to resolve the elution, ion mobility, and mass spectra profiles of the individual chemical constituents present in the simultaneously analyzed microplastic samples according to a bilinear factor decomposition model. The link among these three types of profiles is obtained directly without needing further processing and allowing their annotation and possible identification by comparison with known standard values or with those stored in public databases. This possible identification is improved when simultaneous acquisition of MS2 signals is also performed (see below).

#### Data acquisition-parallel accumulation serial fragmentation

PASEF is a scan mode implemented in the timsTOF Pro instrument, which increases the sequencing speed of MS2 measurements by synchronizing the ion precursor selection with the IM separation without losing sensitivity [24]. Ions are accumulated in parallel, which allows the selection of multiple precursor ions during a single IM scan due to the extremely rapid switching of the quadrupole between precursors on a millisecond time scale [24]. The PASEF method leads to less complex MS2 spectra (previous IM separation) and higher MS2 scan rates [5, 25]. When working with DDA, the same precursor ion is fragmented several times in consecutive scans, generating redundant MS2 spectra. To overcome this limitation, an MS2 spectral dereplication was performed as described in the literature [4]. In addition, all MS2 spectra were binned based on the 3D characteristics of their precursor ions (*m/z*, retention time, and ion mobility values). Within each bin, 3D distances were calculated by combining similarities in precursor ion mobility value, retention time, and MS2 spectral profiles. Finally, a hierarchical cluster analysis (HCA) was performed using these distances to obtain specific MS2 clusters within each bin. Thus, the MS2 spectrum presenting the highest intensity was finally selected to represent the cluster. A more detailed description of this MS2 binning and selection process can be found elsewhere [4]. As a result of the MS2 dereplication process described above, data matrices containing MS1 and MS2 information differ in the number of retention times (rows). In this work, MS1 and MS2 data analyses were performed separately using the ROIMCR method described above. In a postprocessing step, MS1 and MS2 signals of the same component were linked by interpolation using their elution times and precursor ion information (*m/z*), with a retention time tolerance of 0.05–0.2 min applied for signal matching. Thus, a direct link between MS1–MS2 pair spectra of each resolved compound is obtained, preserving their spectral and ion mobility data accuracy.

#### CCS calculation

Reduced ion mobility coefficients (*K*_0_) were then used to obtain the CCS values for each MCR resolved component, enhancing their identification/annotation. CCS is a reproducible physicochemical property of the analyte under defined experimental conditions, as described by the Mason-Schamp equation (Eq. 1) [2].

5 $$\mathrm{C}\mathrm{C}\mathrm{S} (\Omega )= \frac{3}{16}\sqrt{\frac{2\pi }{\mu {k}_{b}T}}\frac{ ze}{{N}_{0}{K}_{0}}$$

where *e* is the elementary charge of an electron, *k*_*b*_ is the Boltzmann constant, *μ* is the ion reduced mass, *N*_0_ is the Loschmidt constant, *T* is the drift region temperature, and *z* is the ion charge. However, this equation was based on oversimplified premises (i.e., consideration of missing low field conditions and that the ion and the neutral molecule are of the same relative size and mass) [26]. In practice, when, as in this work, the trapped ion mobility acquisition method is used, CCS determination is experimentally derived by building a linear regression calibration model with well-characterized ions measured under the same analytical conditions [27], as previously detailed. Therefore, the reported CCS values correspond to experimentally determined CCS values obtained through calibration. The CCS-Predict Pro (Bruker Daltonics, Bremen, Germany) software, implemented in MetaboScape (MetaboScape 2023b, Bruker Daltonics, Bremen, Germany), was used to generate reference CCS values and compare the values with those obtained by the ROIMCR method.

#### Plastic additives identification

**Fig. 1:**
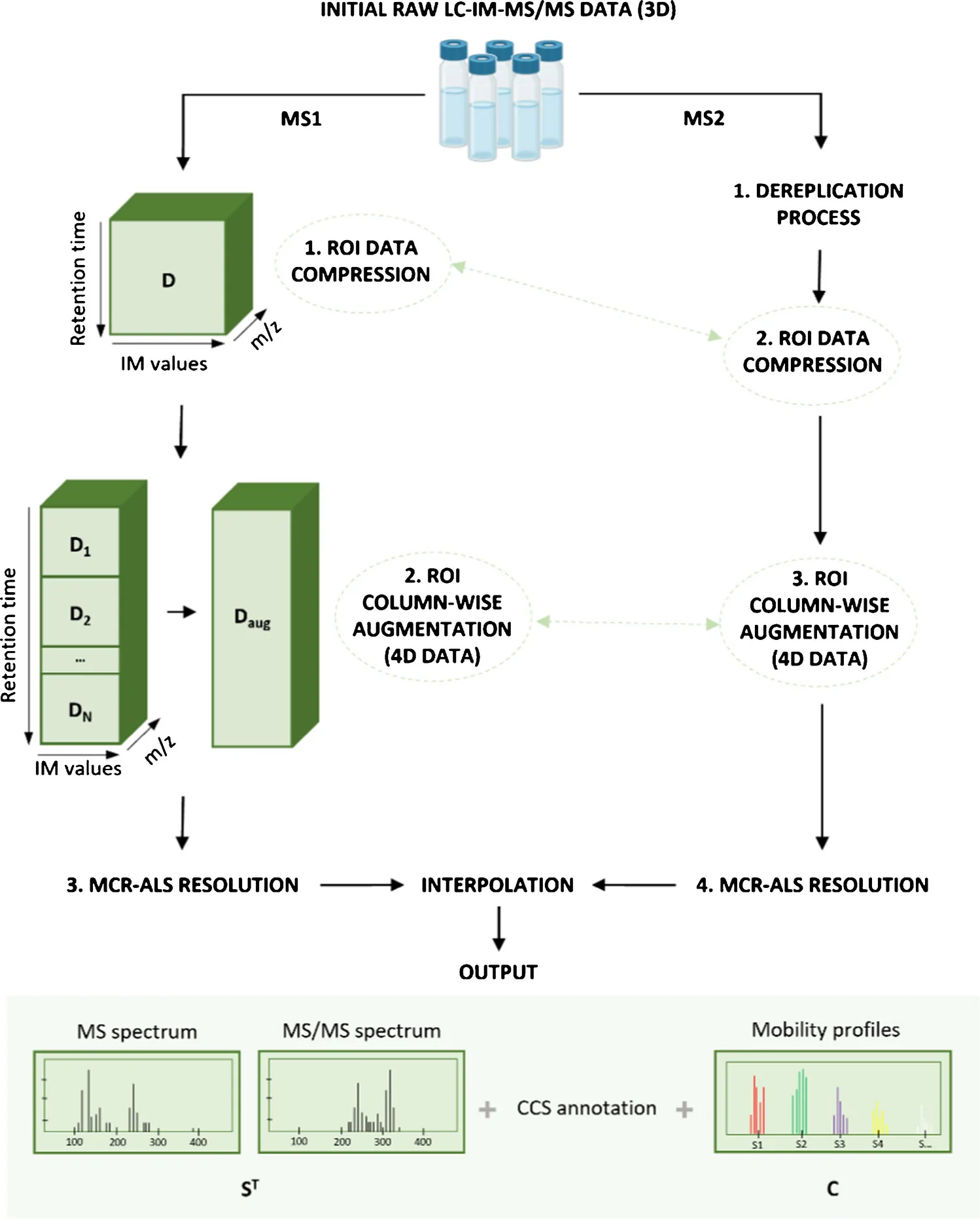
Schematic overview of the proposed ROIMCR workflow for the analysis of LC-IM-MS data, providing, as output, for each resolved component, MS and MS2 spectra, experimentally determined CCS value, and ion mobility profile for compound annotation

Preliminary identification of plastic additives in the PE standards was performed by analyzing *m/z* MS1 signals in the mass spectra of the ROIMCR-resolved components. In a second step, MS2 signals and experimentally determined CCS values were used to improve identification and confirmation reliability. CCS values were matched against the vendor-provided Bruker CCS database, and values falling a ± 2% tolerance were considered acceptable for compound identification, as reported in the literature [6, 28]. Compounds were confirmed when both MS2 fragmentation ions and CCS values matched the reference within the specified criteria. An overview of the proposed LC-IM-MS data processing workflow based on ROIMCR is shown in Fig. 1. Finally, a comparative data processing and identification workflow was carried out using MS-DIAL (version 5.5260323), a widely used open-source software for non-targeted analysis of mass spectrometry data [29]. The LC-IM-HRMS/MS datasets were processed following standard feature detection, deconvolution, and alignment procedures implemented in MS-DIAL. The parameters were selected to closely match those used in the ROI analysis, with minor adjustments such as intensity threshold of 528 and mass tolerance of 0.01 Da (MS1) to increase sensitivity, and the same database was used for identification. Additional details are provided in Table S4 in the Supporting Information, including peak picking and alignment settings. This approach was used as a reference to evaluate the performance of the proposed ROIMCR method in terms of feature resolution and data interpretation.

## Results and discussion

This results section is divided into three parts. In the first part, the results of the individual LC-IM-MS analysis of a PE standard leachate (see experimental section dataset 1) are presented. The data were acquired in a single LC-IM-MS run and subsequently extracted and analyzed separately as LC-MS and IM-MS datasets using the ROIMCR procedure, and their possible differences in terms of sensitivity, selectivity, and component resolution were assessed. In the second part, LC-IM-MS1-MS2 datasets of five different-size standard mixture PE spheres (S1, S2, S3, S4, and S5, dataset 2) were simultaneously analyzed by the proposed ROIMCR method to evaluate differences in the presence of additives in the standards. Finally, in the third part, LC-IM-MS1-MS2 datasets obtained from KIT #205 (K1 and K2, dataset 3) PE samples were analyzed by ROIMCR to test its performance for the analysis of unknown samples.

### Comparative ROIMCR analysis of LC-MS and IM-MS data from one PE standard microsphere sample (Data Example 1)

The S1 sample was analyzed in detail (dataset 1) using both LC-MS1 and ion mobility, IM-MS1, to compare their respective analytical performance. The ROI procedure was applied separately to MSroi and IMroi data matrices obtained using LC-MS and IM-MS data acquisitions, respectively. Table 1 summarizes the ROI parameters optimized in each case. For the ROI analysis, a 0.85% of the maximum MS intensity threshold value was chosen in both cases after preliminary data inspection, providing an optimal compromise between signal retention and noise reduction across samples. This value was selected after evaluating a range of thresholds (0.1–1% of the maximum signal intensity): lower thresholds (below 0.5%) introduced non-informative noise-related components with diffuse chromatographic profiles and non-reproducible mass spectra, while higher thresholds (above 1%) led to the loss of low-abundance analytes. This intensity threshold value prevents noisy LC-IM-MS signals from being selected for analysis. The value of 8 ppm was selected as mass error tolerance, considering the mass accuracy of the mass spectrometer used (time of flight) as the *m/z* admissible mass deviation error is associated with the MS instrument and its spectral resolution. A minimum number of 6 data point occurrences at the same *m/z* values in consecutive scans were considered to define a ROI, which depends on the chromatographic system used and the scanning speed of the detector. This parameter was set according to the chromatographic peak width and scan rate of the instrument. In this case, the 0.32-s duty cycle and typical chromatographic peak width at half-maximum of 3–5 s, a minimum of 6 consecutive occurrences ensures that chromatographic signals are retained while noise signals are excluded.

**Table 1:** Summary of ROIMCR-ALS parameters and results, including number of components, *R*^2^, and lack of fit (Dataset 1)

**ROI parameters and results**^**a**^					
	Signal threshold	*m/z* error (ppm)	Minimum number of occurrences	Number of *m/z* ROIs	
Original data	6200	8	6	153	
**MCR-ALS parameters and results**^**b**^					
	ALS constraints	ALS initials	Number of components	*R*^2^ (%)	lof (%)
MSroi	Nn, norm	C	9	99.86	0.82
MSroi_IM	Nn, norm	C	20	97.68	7.76

^a^Optimized ROI parameters: signal threshold (between 0.1% and 1% of the maximum signal intensity); *m/z* error: mass accuracy considered according to the LC-MS instrument used, in this case 8 ppm; minimum number of occurrences: minimum number of signals to consider to form a ROI defining a chromatographic peak; number of *m/z* ROIs finally selected

^b^MCR-ALS parameters: ALS constrains, non-negativity of resolved elution and mass spectra profiles (nn) and mass spectra normalization to maximum MS signal intensity equal to one (norm); ALS initials, purest elution concentrations profiles (**C**); number of components resolved by MCR-ALS; *R*^*2*^, explained data variance (Eq. (3)); *lof*, lack of fit with the considered number of components (Eq. (4))

As a result, two compressed data matrices (MSroi and IMroi) were obtained with 310 *m/z* ROI values (chromatograms as columns in the data matrix) and 1296 retention times (ROI mass spectra as rows in the data matrix). Large differences in the signal intensities were observed between the MSroi and IMroi data, reflecting their different nature and scales (Figure S1). MSroi and IMroi individual data matrices were processed with MCR-ALS chemometric analysis to resolve separately the elution and ion mobility profiles and pure MS spectra of the sample constituents.

**Fig. 2:**
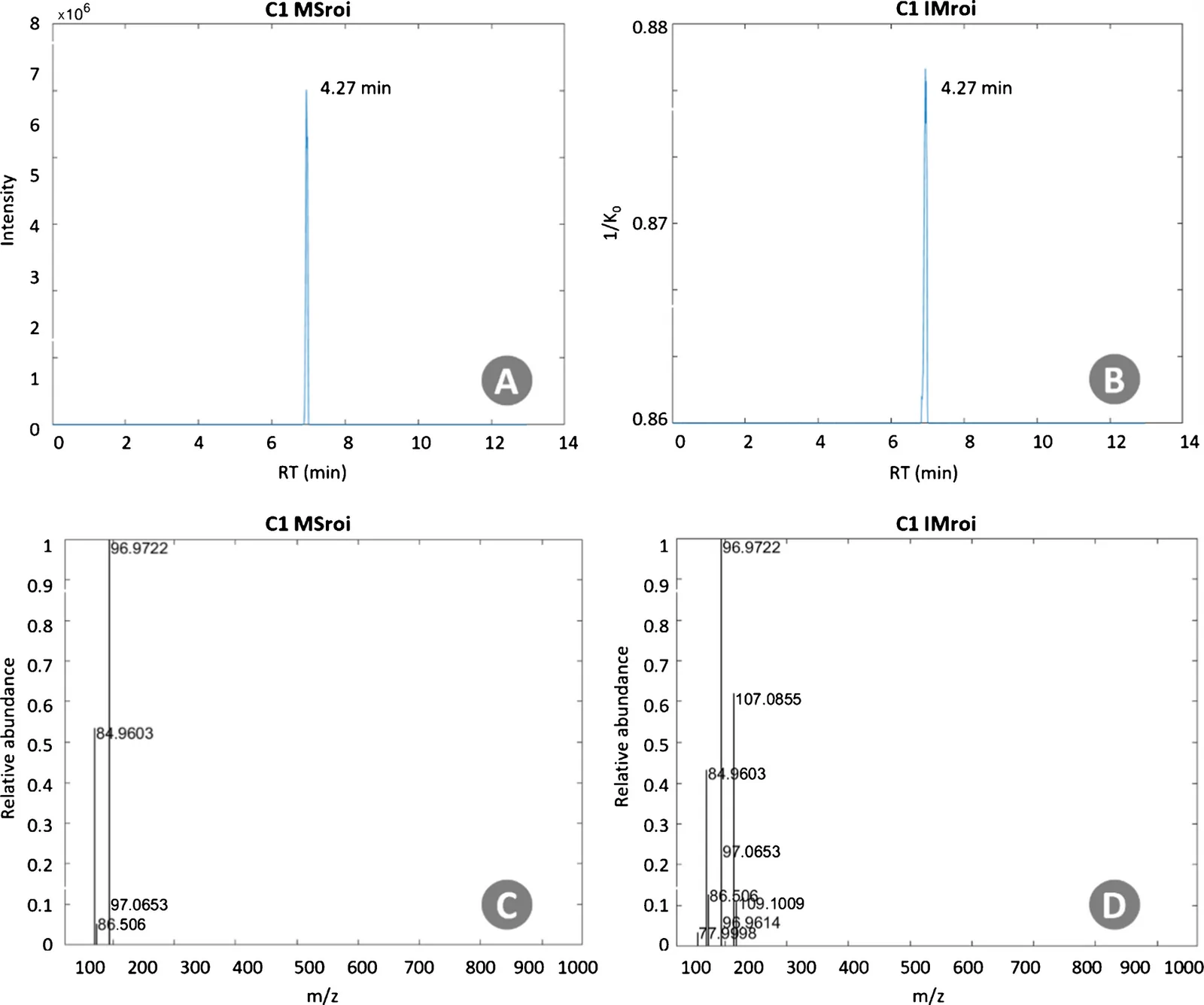
ROIMCR-resolved component 1 (C1) showing elution and mobility profiles (**A** and **B**, respectively) and mass spectral profiles obtained from LC-MS (**C**) and LC-IM-MS (**D**)

**Fig. 3:**
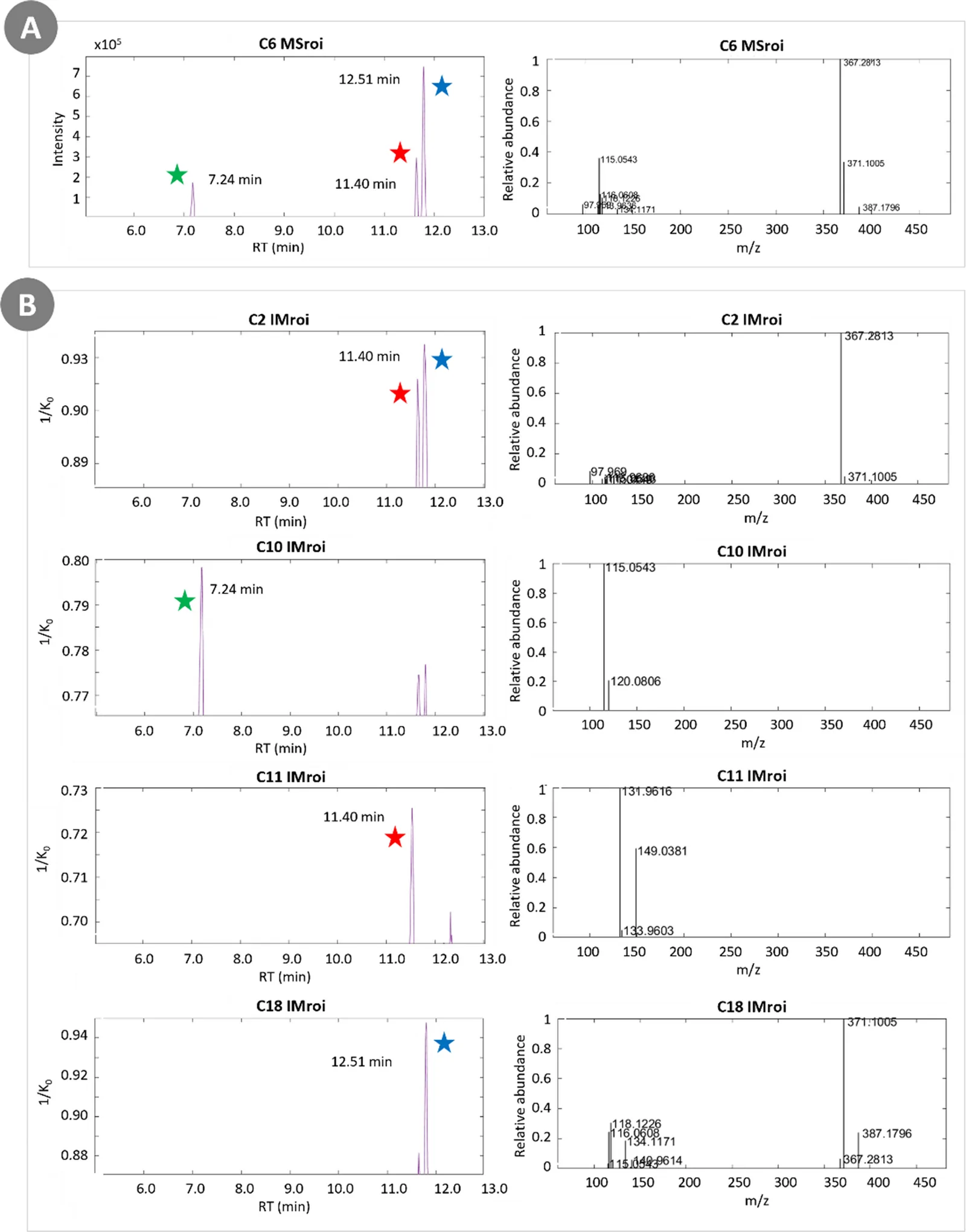
Comparison of MCR resolved components obtained from LC-MS (**A** MSroi) data and from IM-MS (**B** IMroi) data. Left panels show the resolved elution/mobility profiles as a function of chromatographic scan index, while right panels show the corresponding mass spectra profiles. In IM-MS, components C2, C10, C11, and C18 are individually distinguished based on their ion mobility profiles. In contrast, these components are merged and appear as a single component (C6) in the MS dataset (MSroi)

The number of components finally proposed for the MCR-ALS was 9 (MSroi) and 20 (IMroi), respectively, explaining 99.86% and 97.68% of the total data variances (Table 1), respectively. The final number of components was obtained according to the singular value decomposition (SVD) of the analyzed data matrices, as described in Text S1. The correspondence between MSroi and IMroi MCR components was established using an interpolation-based matching approach, in which IMroi-resolved components were mapped onto MSroi components based on retention time, elution profiles, and *m/z* information. Two different patterns were observed when comparing the results from both data acquisitions (datasets). First, there were common components resolved in both cases. For instance, MCR components (C) C1 in MSroi and C1 in IMroi correspond to the same chemical compound as they have the same elution profile, retention time, and mass spectra (Fig. 2). Second, several single MCR components in MSroi correspond to more than one component in IMroi, which is reasonable since IM-MS enhances MS selectivity, sensitivity, and resolving power [28]. For instance, in IMroi, MCR components C2, C10, C11, and C18 in IMroi are resolved just as C6 in MSroi (Fig. 3). The profiles shown in Fig. 3 correspond to the different ROIMCR-resolved components obtained from the IMroi data matrix. These profiles represent the mobility-resolved component obtained after MCR procedure. The *x*-axis of the figure represents the elution coordinate (scan index), while the *y*-axis corresponds to the reduced ion mobility values associated with the elution. The small variations in 1/*K*_0_ across scans observed in raw IM-MS data are resolved as different mobility profiles by using ROIMCR. Other examples were observed in MCR components C15 and C17, resolved in a single MSroi component C4 (see Figure S2A) or components C5 and C19 in IMroi, resolved as C7 in MSroi (Figure S2B).

**Fig. 4:**
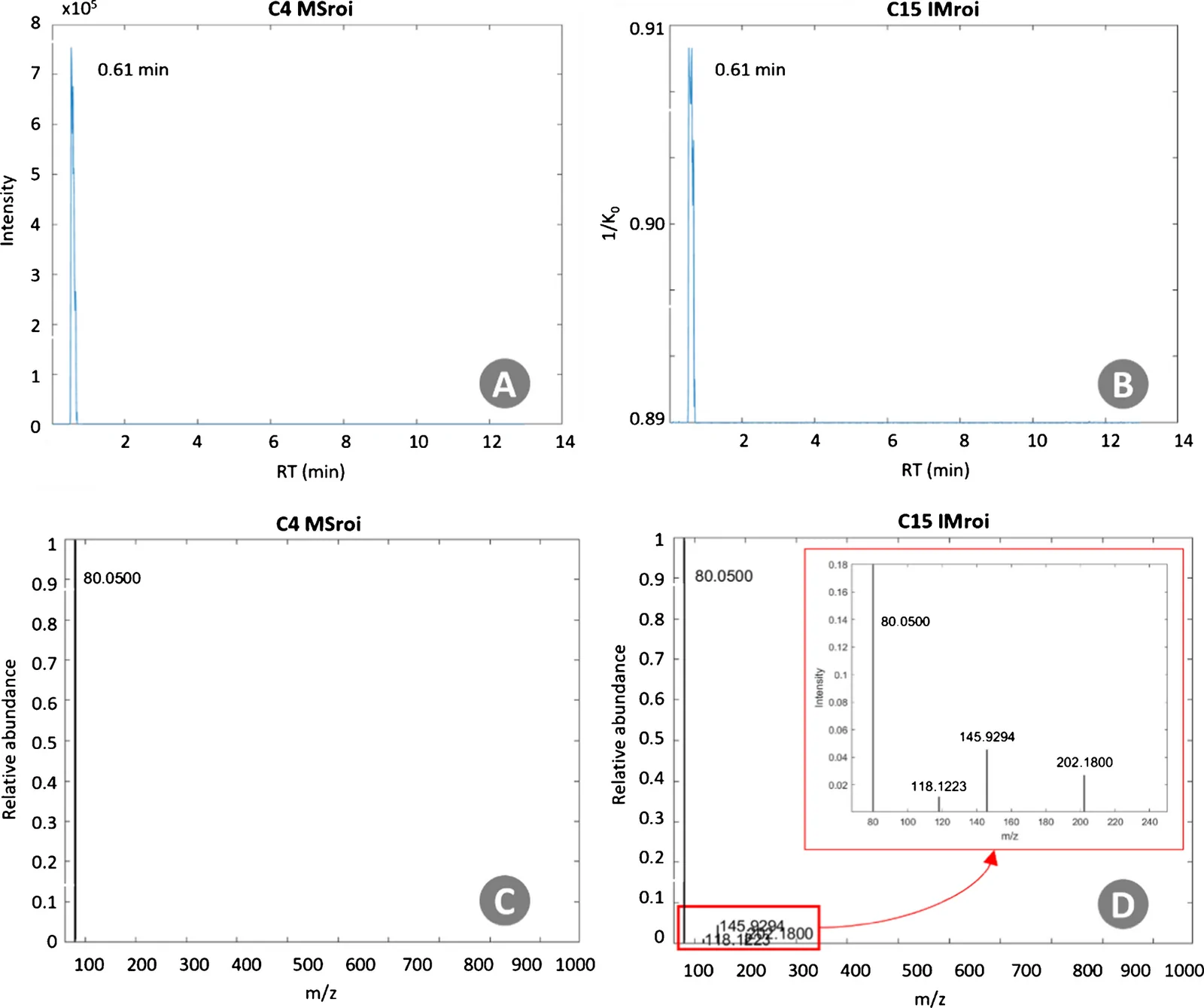
Comparison of ROIMCR-resolved components obtained from LC-MS (MSroi) and IM-MS (IMroi) data. Upper panels show LC-MS (**A**) and IM-MS (**B**) elution profiles (scan index) of resolved component C6, while lower panels display their corresponding mass spectra in the two modes (**C** and **D**, respectively). IM-MS results (right) show enhanced sensitivity and improved signal-to-noise ratio, allowing clearer detection of additional MS signals (i.e., at *m/z* 118.1223, 145.9294, 202.1800), which were not resolved in LC-MS (left) where only *m/z* 80.500 is detected

We can conclude, therefore, that although MSroi and IMroi data are obtained from the same data file, IM-MS showed higher sensitivity and resolving power compared with traditional LC-MS analysis (Fig. 4). Quantitatively, the number of resolved MCR components increased from 9 (MSroi) to 20 (IMroi), representing a 122% improvement in component resolution when full ion mobility profiles are exploited instead of LC-MS data alone. This improvement is directly attributable to the ability of IM to distinguish species with similar *m/z* values and overlapping retention times based on differences in their ion mobility. While in the LC-MS analysis, ions are mainly resolved based on their elution times and *m/z* values, in the IM-MS, ions with similar *m/z* eluting at the same times can be further distinguished by their different ion mobilities, improving resolution power and signal-to-noise ratio (S/N), and reducing peak overlapping. As previously stated, this enhanced resolution leads to a higher number of component resolutions when working with IM. Therefore, clearer and more comprehensive spectra are obtained due to increasing sensitivity and the reduction of noise interferences. As shown in Fig. 4, IMroi data also exhibits a clearly improved S/N ratio compared to MSroi, enabling the detection of additional mass signals (at *m/z* 118.1223, 145.9294, and 202.1800) that were not observable in LC-MS mode. Taken together, the 122% increase in resolved components, the resolution of coeluting isobaric species (e.g., C6 in MSroi resolved as C2, C10, C11, and C18 in IMroi), and the improved S/N all provide quantitative evidence that IM integration should not be based solely on the availability of CCS values, but on the exploitation of full ion mobility profiles, which provide additional selectivity and enable better resolution of coeluting isobaric species that remain unresolved in LC-MS data.

### Simultaneous ROIMCR analysis OF LC-IM-MS1-MS2 data from five PE standard microsphere samples (S1, S2, S3, S4, S5, Data Example 2)

In this second analysis, the ROIMCR method was applied to the simultaneous analysis of five PE standard samples with different MP sizes (S1, 45–53 µm; S2, 106–125 µm; S3, 300–355 µm; S4, 500–600 µm; and S5, 850–1000 µm) (dataset 2). A column-wise (vertically concatenated) augmented MSroi (MS1) data matrix was built containing the common and uncommon *m/z* ROI values obtained in the analysis of all individual datasets (S1–S5). Prior to data matrix augmentation, each individual data matrix was scaled by its first singular value, following the procedure described by Golub (1970) [21]. This scaling preserves the weight of each matrix and ensures equal contribution of every individual data matrix to the final augmented data matrix. IMroi augmented data matrix contained a total of 5 × 1295 = 6475 rows by 557 *m/z* ROI values (columns). The total ion chromatograms (TICs) at all retention times are presented graphically in Figure S3A. In this case, the total number of MCR components proposed was 30, explaining 95.1% of the data variance. From the 30 initially proposed MCR components, eight were associated with instrumental background and other possible systematic noise sources, giving non-reliable mobility profiles and mass spectra, and they were not further considered. The same procedure was followed for the MS2 data obtained by DDA, building up an IMroiMS2 matrix having 13,730 rows (2960, 2885, 2670, 2738, and 2477 retention times) and 672 columns (ROI values) (Figure S3B). Due to the high number of signals in DDA MS2 data, a significantly larger number of MCR components were initially proposed, up to 275, which explained 98.6% of the total data variance. This difference compared to the number of components resolved from MS1 data is due to the higher complexity of DDA MS2 data compared to MS1 data, where each precursor in MS1 produces multiple fragment ions in MS2. As in the MS1 analysis, non-negativity constraints on elution and spectral profiles, as well as spectral normalization (equal height), were applied during the MCR-ALS analysis of MS2 data. MCR IM-MS1 and IM-MS2 resolved elution profiles were synchronized by interpolation, enabling the association of the corresponding mass spectra for each component. Moreover, CCS values were calculated from IM-MS2 as an extra indicator for compound identification.

The NIST Tandem Mass Spectral Library 2023 release (NIST23) contains 2.4 million spectra, including 51,501 compounds and 399,267 precursor ions [30]. This NIST library, developed for LC analysis, was selected for the identification of the plastic additives present in the samples. Among the 22 components showing good chromatographic and spectral features (i.e., well-defined chromatographic peak shape, consistent ion mobility profile, and high signal-to-noise mass spectra), seven were directly identified as plastic additives, showing the potential use of the proposed method to characterize unknown compounds in complex mixtures. Table 2 summarizes the identified compounds, listing the names, component number, CAS numbers, experimental and reference CCS values, molecular formulas, molecular weight, and potential applications. Identification confidence was assigned following the Schymanski et al. (2014) framework, where compounds confirmed by accurate MS1 mass match (< 8 ppm) and MS2 spectral library match (NIST23 score > 700) were classified as Level 2 (probable structure). Additionally, ΔCCS values below 2% were considered acceptable for tentative identification, consistent with literature recommendations [6, 28]. 1,3:2,4-bis(3,4-Dimethylbenzylidene) sorbitol (DMDBS) is a sorbitol-derived nucleating agent widely used to regulate polymer crystallization and enhance material performance by increasing their nucleation density [31]. 2,2′-(Tridecylazanediyl)bis(ethan-1-ol) is a long-chain ethoxylated amine traditionally used as a migrating antistatic, preventing the build-up of static electricity in the polymer by attracting water molecules [32]. Alkanox 240 is a well-known commercial phosphate antioxidant, which has been previously found in other investigations studying PE and using isopropanol as a solvent [33]. Phthalates (i.e., butyl 2-ethylhexyl phthalate) and siloxanes (i.e., decamethylcyclopentasiloxane) are extensively utilized as plastic additives owing to their exceptional and multifunctional plasticizing effects. Phthalates exhibit strong plasticizing properties, enhancing material flexibility, durability, and overall stability [34], while siloxanes are used to enhance the mechanical performance of polymers, especially increasing the scratch resistance of plastic surfaces [35, 36]. Finally, erucamide is a lubricant used during manufacturing, having both internal and external lubricating functions, and triphenylphosphine oxide (TPPO) is a flame retardant that avoids or delays combustion, melt collapse, and smoke generation [37, 38]. It should be noted that erucamide presented a ΔCCS deviation of 1.9%, close to the selected cutoff. Although a stricter threshold (e.g., ± 1.5%) would exclude this feature based solely on CCS agreement, its annotation was additionally supported by accurate mass matching of the precursor ion, MS/MS spectral similarity, its known use as a slip additive in polyethylene materials, and its independent identification using MS-DIAL. Therefore, erucamide was retained as a plausible tentative identification within the applied non-target screening workflow. The comparative evaluation with MS-DIAL showed that 4 out of the 7 compounds identified using the ROIMCR approach were also detected using the conventional MS-DIAL workflow (reference matched, total score > 800), including the identification of Alcanox 240, butyl 2-ethylhexyl phthalate, erucamide, and TPPO. The presence of the other compounds was not even suggested (only MS1 spectrum confirmed). This result indicates a good level of agreement between both approaches while also highlighting the added value of ROIMCR. In particular, ROIMCR enabled a more effective exploitation of the ion mobility dimension and improved the resolution of overlapping signals, which contributed to an increased number of confidently identified compounds. All these results obtained in the analysis of five PE leachate samples (S1, S2, S3, S4, and S5) confirmed the adequacy of the ROIMCR method for the huge IM-MS data analysis.

**Table 2:** Tentative identification of each ROIMCR component (alphabetic order) obtained from the analysis of PE standard microsphere samples (S1, S2, S3, S4, S5, data set example 2) including name, CAS number, molecular formula, precursor type, experimentally determined CCS values (obtained from ROIMCR analysis), reference CCS values (predicted from the vendor Bruker software), molecular formula, and potential application of the chemical compound

**Name**	**RT (min)**	**CAS number**	**Molecular formula**	**Precursor type**	**Experimental CCS value (Å)**	**Reference CCS value (Å)**	**│∆CCS│ (%)**	**Application**
DMDBS	7.31	81541-12-0	C_22_H_26_O_6_	[M + H]^+^	185.3	184.3	0.5	Nucleating agent
2,2′-Tridecylazanediylbis(ethan-1-ol)	13.26	18312-57-7	C_17_H_37_NO_2_	[M + H]^+^	179.4	180.3	0.5	Antistatic and dispersive agent
Alkanox 240	14.62	95906-11-9	C_42_H_63_O_4_P	[M + H]^+^	250.9	253.2	0.9	Antioxidant coating and adhesive
Butyl 2-ethylhexyl phthalate	12.59	85-69-8	C_20_H_30_O_4_	[M + H]^+^	184.0	184.3	0.2	Plasticizer
Decamethylcyclopentasiloxane	14.89	541-02-6	C_10_H_30_O_5_Si_5_	[M + H]^+^	170.9	171.7	0.5	Surfactant
Erucamide	13.15	112-84-5	C_22_H_43_NO	[M + H]^+^	195.0	198.9	1.9	Slip additive
TPPO	14.30	791-28-6	C_18_H_15_OP	[2M + H]^+^	159.5	160.1	0.4	Flame retardant

### ROIMCR analysis of LC-IM-MS1-MS2 data from PE microplastic additive samples (KIT #205 SAMPLES K1, K2, AND K3, Data Example 3)

Firstly, datasets from individual LC-IM-MS1-MS2 analysis of samples K1, K2, and K3 (dataset 3) were scaled by their first singular value. ROI simultaneous analysis of these samples produced an IMroiMS1 data matrix with a total of 3 × 1296 = 3888 rows and 835 columns (*m/z* ROI values), see Supplementary Information, Figure S4A). Non-negativity constraints in elution and spectral profiles and spectral normalization (equal height) were applied during MCR-ALS. Twenty-six components were resolved in this case, explaining 95.3% of the total data variance. Seven non-informative components, including instrumental and background noise, were filtered out based on the unreliable shape of their spectral and mobility profiles. MS2 DDA data provided an IMroiMS2 augmented data matrix, with 8902 rows (3921, 2573, and 2408 retention times, respectively for K1, K2, and K3 samples) and 315 columns (*m*/*z* ROI values) (see Figure S4B). As explained for data example 2, MS2 DDA data exhibit higher complexity than MS1 due to the generation of multiple fragment ions per precursor, which increases the number of detected signals and consequently the dimensions of the IMroiMS2 augmented data matrix. The MCR-ALS analysis of this augmented data matrix using 97 MCR components explained 99.4% of the data variance. Using a similar procedure as in the previous data example, synchronization between MCR components through their elution times and *m/z* MS1 and MS2 precursor information allowed the calculation of the corresponding CCS values, and ΔCCS values below 2% were considered acceptable for tentative identification.

Seven different unknown plastic additives were identified in the PE samples from Kit #205 (Table 3). Phthalates (bis-(7-methyloctyl) phthalate, diisobutyl phthalate, and mono-2-ethylhexyl phthalate) and siloxanes (decamethylcyclopentasiloxane and hexamethylcyclotrisiloxane) were identified as plasticizers in these PE samples. Hexaethylene glycol (a low-weight polyethylene glycol) was also present in the samples, probably due to its use as a plasticizer or as an internal lubricant during PE extrusion, which reduces its die pressure and melt viscosity [39]. Finally, as observed in the previous PE microsphere samples, Alkanox 240 was also detected in this sample case.

**Table 3:** Tentative identification of each ROIMCR component (in alphabetic order) obtained from home-made PE microplastic additive samples (from Kit #205 samples K1, K2, and K3, data example 3) including name, CAS number, molecular formula, precursor type, experimentally determined CCS values (obtained from ROIMCR analysis), reference CCS values (predicted from the vendor Bruker software), molecular formula, and potential application of the chemical compound

Name	RT (min)	CAS number	Molecular formula	Precursor type	Experimental CCS value (Å)	Reference CCS value (Å)	│∆CCS│ (%)	Application
Bis-(7-methyloctyl) phthalate	13.13	20548-62-3	C_26_H_42_O_4_	[M + H]^+^	211.7	210.3	0.7	Plasticizer
Decamethylcyclopentasiloxane	14.89	541-02-6	C_10_H_30_O_5_Si_5_	[M + H]^+^	172.9	171.7	0.7	Surfactant
Diisobutyl phthalate	14.76	84-69-5	C_16_H_22_O_4_	[M + H]^+^	163.4	166.1	1.6	Plasticizer
Hexaethylene glycol	0.78	2615-15-8	C_12_H_26_O_7_	[M + H]^+^	164.0	167.0	1.8	Surfactant
Hexamethylcyclotrisiloxane	14.38	541-05-9	C_6_H_18_O_3_Si_3_	[M + H]^+^	151.3	149.0	1.5	Surfactant
Mono-2-ethylhexyl phthalate	11.82	4376-20-9	C_16_H_22_O_4_	[M + H-C_8_H_18_O]^+^	165.5	167.3	1.1	Plasticizer
Alkanox 240	14.62	95906-11-9	C_4_H_63_O_4_P	[M + H]^+^	250.9	253.2	0.9	Antioxidant coating and adhesive

## Conclusions

In the present study, the ROIMCR approach is applied for the first time in the analysis of LC-IM-MS data. Ion mobility profiling showed the resolution of a greater number of components than liquid chromatography, because of the effective discrimination of isomeric species, enhanced mass spectral sensitivity, and clearer mass spectra profiles. Therefore, ROIMCR proved to be an effective and valuable chemometric tool to handle the high dimensionality and complexity of LC-IM-MS datasets by explicitly modeling ion mobility profiles. Unlike most IM-MS data processing workflows, which primarily exploit ion mobility using only CCS descriptor values for additional chemical species identification, the ROIMCR strategy integrates ion mobility as a new structured analytical dimension within the multivariate model. The proposed procedure was successfully applied to a set of pristine PE standards and PE leachates containing unknown plastic additives, allowing the identification of different phthalates, siloxanes, and organophosphates, among others. Overall, our results highlighted the advantages of using the proposed ROIMCR chemometric method in the processing of IM-MS data, not only for an enhanced comprehensive analysis of complex and information-rich datasets, but also for improving non-targeted identification of complex mixtures, where IM plays a key role in the separation and structural characterization of unknown analytes. Future studies should explore the applicability of ROIMCR to more complex scenarios, including aged or weathered plastics, environmental leaching experiments under realistic conditions, and real-world samples such as sediments, biota, and human matrices affected by plastic exposure.

## Supplementary Information

Below is the link to the electronic supplementary material.

Supplementary file1 (DOCX 1.43 MB)

## Data Availability

All data have been provided in the manuscript and the supplementary information material.
