# Supplementary material for: Regions of interest multivariate curve resolution for liquid chromatography with ion mobility high-resolution tandem mass spectrometry: analysis of plastic additives
Source: Anal Bioanal Chem. 2026 Jun 22;418(18):5857–70. doi: 10.1007/s00216-026-06632-w (PMC13541791; doi:10.1007/s00216-026-06632-w)
Supplement: Supplementary file 1 [file 216_2026_6632_MOESM1_ESM.docx]

**Supporting Information**

**Regions of interest multivariate curve resolution for liquid chromatography with ion mobility high resolution tandem mass spectrometry: analysis of plastic additives**

Ana Torres-Agullo^1,2^, Roma Tauler^1^, Silvia Lacorte^1^, Tytus D. Mak^3^, Yamil Simón-Manso^3^

^1^Institute of Environmental Assessment and Water Research of the Spanish Research Council (IDAEA-CSIC). Jordi Girona 18-26, 08034 Barcelona, Spain.

^2^Department of Chemical Engineering and Analytical Chemistry, Universitat de Barcelona, Diagonal 645, 08028 Barcelona, Spain.

^3^Mass Spectrometry Data Center, Biomolecular Measurement Division, National Institute of Standards and Technology (NIST), Gaithersburg, MD 20899, USA.

**List of abbreviations and acronyms**

| Abbreviation | Definition |
| --- | --- |
| ALS | Alternating Least Squares |
| C | MCR Resolved Component |
| CCS | Collision Cross Section |
| DDA | Data-Dependent Acquisition |
| D_aug_ | Augmented data matrix |
| GC-HRMS | Gas Chromatography–High Resolution Mass Spectrometry |
| HCA | Hierarchical Cluster Analysis |
| HRMS | High-Resolution Mass Spectrometry |
| IM | Ion Mobility |
| IM-MS | Ion Mobility–Mass Spectrometry |
| IMroi | Ion mobility Regions of Interest data matrix |
| K₀ | Reduced ion mobility coefficient |
| LC | Liquid Chromatography |
| LC-IM-MS | Liquid Chromatography–Ion Mobility–Mass Spectrometry |
| LC-MS | Liquid Chromatography–Mass Spectrometry |
| LC-MS/MS | Liquid Chromatography–Tandem Mass Spectrometry |
| lof | Lack of fit |
| MCR | Multivariate Curve Resolution |
| MCR-ALS | Multivariate Curve Resolution–Alternating Least Squares |
| MP | Microplastics |
| MS | Mass Spectrometry |
| MS1 | Full-scan mass spectrometry |
| MS2 | Tandem mass spectrometry |
| MSroi | Mass spectrometry Regions of Interest data matrix |
| PASEF | Parallel Accumulation–Serial Fragmentation |
| PE | Polyethylene |
| POPs | Persistent Organic Pollutants |
| QA/QC | Quality Assurance / Quality Control |
| ROI | Regions of Interest |
| ROIMCR | Regions of Interest Multivariate Curve Resolution |
| S/N | Signal-to-noise ratio |
| SVD | Singular Value Decomposition |
| TIC | Total Ion Chromatogram |

**TEXT S1. CHEMOMETRIC METHODS**

**REGIONS OF INTEREST (ROIs)**

LC-IM-MS provides high-dimensional datasets containing *m/z*, retention time, mobility, and MS1 or/and MS2 spectral information, which requires massive storage and processing. Since the paired *m/z*, MS signal intensity, and mobility values are not equidistant in the *m/z* direction, a preliminary data pretreatment and compression step is required to transform the LC-IM-MS data to computationally manageable formats (data matrices). The ROI procedure was selected as a preprocessing methodology to handle the large and complex LC-IM-MS data sets since it allows their compression and data matrix transformation without any loss of their spectral accuracy [20]. Using this procedure, specific regions of high-density data points (referred to as ROIs) are identified on the raw LC-IM-MS spectral scans according to specific predefined criteria, including a signal intensity threshold value (estimated to be higher than the instrumental noise level, usually between 0.01 % to 1 % of the maximum measured signal intensity), the mass accuracy (*m/z* deviation error), and the minimum number of measurements to be included in one ROI. When working with MS data, the ROI procedure gives two main outputs: a vector including the list of ROI *m/z* values (mzroi) and an MSroi data matrix containing the MS signal intensities of the selected ROIs at all retention times. When working with LC-IM-MS data, an additional output is generated: a new data matrix (IMroi) containing the mobility values at each *m/z* ROI values (columns) and retention times (rows). This provides a three-dimensional (mode, way) data set. The MCR-ALS method can be applied to simultaneously analyze these ROI data sets, as discussed in the following section.

**MULTIVARIATE CURVE RESOLUTION – ALTERNATING LEAST SQUARES (MCR-ALS)**

When applied to LC-MS data, Multivariate Curve Resolution – Alternating Least Squares (MCR-ALS) resolves the elution and spectra profiles of the individual components in the analyzed samples according to the bilinear factor decomposition model described by Equation 1.

$\mathbf{D=C}\mathbf{S}^{\mathbf{T}}\mathbf{+E}$	(1)

Where matrix **D** (I, J) corresponds to MSroi data with the recorded mass spectra at all chromatographic retention times (i = 1, …., I) as the rows and with the chromatograms (elution profiles) at the ROI selected *m/z* values (j = 1, …, J) as the columns. The MCR bilinear decomposition gives two factor matrices, **C** (I, N) with the chromatographic elution profiles of the individual components (n = 1, …, N) and **S^T^** (N, J), with the mass spectra of these resolved components (Equation 1). **E** (I, J) contains the residuals describing the data variance that the MCR bilinear model does not explain. In the case of IM-MS data, the factor matrix **C** (I, N) will contain the mobility profiles of each component (n = 1, …, N), while factor matrix **S^T^** remains the mass spectra of these components.

When multiple chromatographic/mobility runs (k = 1, …, K) are obtained, individual ROI data matrices can be concatenated vertically, giving a new column-wise augmented MSROI data matrix (**D_aug_**), which can be analyzed simultaneously by MCR-ALS assuming an extended bilinear model according to the following Equation 2:

$\mathbf{D}_{\mathbf{aug}}\mathbf{=}\mathbf{C}_{\mathbf{aug}}\mathbf{S}^{\mathbf{T}}\mathbf{+}\mathbf{E}_{\mathbf{aug}}\boldsymbol{\to}\left[ \begin{matrix} \mathbf{D}_{\mathbf{1}} \\ \mathbf{D}_{\mathbf{2}} \\ \begin{matrix} \mathbf{.} \\ \begin{matrix} \mathbf{.} \\ \mathbf{D}_{\mathbf{K}} \end{matrix} \end{matrix} \end{matrix} \right]\mathbf{=}\left[ \begin{matrix} \mathbf{C}_{\mathbf{1}} \\ \mathbf{C}_{\mathbf{2}} \\ \begin{matrix} \mathbf{.} \\ \begin{matrix} \mathbf{.} \\ \mathbf{C}_{\mathbf{K}} \end{matrix} \end{matrix} \end{matrix} \right]\mathbf{S}^{\mathbf{T}}\mathbf{+}\left[ \begin{matrix} \mathbf{E}_{\mathbf{1}} \\ \mathbf{E}_{\mathbf{2}} \\ \begin{matrix} \mathbf{.} \\ \begin{matrix} \mathbf{.} \\ \mathbf{E}_{\mathbf{K}} \end{matrix} \end{matrix} \end{matrix} \right]$	(2)

Where **C_aug_** (I x K, N) contains the concentration/elution profiles of the N components identified across the K analyzed samples and **S^T^** represents the corresponding mass spectra of these components. In this equation (Equation 2), the mass spectra in (**S^T^**) are the same for the common constituents in the different chromatographic/mobility runs (individual) data matrices. In contrast, the elution profiles (**C_aug_**) can be different in the different chromatographic runs.

MCR-ALS analysis of **D** (single data set obtained in the analysis of one sample) or **D_aug_** (multiple data sets obtained in the analysis of several samples) matrices are performed using Alternating Least Squares (ALS) iterative optimization. This optimization requires an initial proposal of the number of components explaining all possible data variance sources, i.e., chemical constituents of the sample and other potentially unknown contributions originating from the background, solvent, and mass spectrometer. The number of components can be initially estimated by evaluating the number of the largest singular values (larger than those related to experimental error) derived from the Singular Value Decomposition (SVD) of the analyzed data matrices [21]. A sufficiently large number of components needed to be considered, including sample constituents at low concentrations. This selection was performed considering the amount of explained data variance and the reliability of the resolved component profiles (elution, mobility, and mass spectra). Too many components will not improve the data variances significantly, and the shapes of the additional component profiles will not be physically reliable and too noisy. Once the number of components is selected, an initial estimation of either the elution (**C**) or spectral (**S^T^**) profiles is provided to start the ALS optimization. These initial estimates can be obtained directly from the measured data, either from the more different elution profiles (at particular ROI *m/z* values) or from the more different spectral profiles (at particular retention times), using a procedure like the SIMPLISMA method [22]. Different constraints (i.e., chemical, physical, or mathematical) are applied during the ALS optimization to reduce the ambiguity associated with the MCR bilinear factor decomposition. Due to the high selectivity of LC-MS and LC-IM-MS data, this ambiguity is usually low, and the use of non-negativity constraints for elution and spectral profiles and spectra normalization (their maximum heights equal to one) already provides reliable solutions fitting well the analyzed experimental data, either working with LC-MS or with IM-MS data. Data fitting at convergence was evaluated by the percentage of explained data variance (R^2^) and by the data lack of fit (lof) values, as shown in Equations (3) and (4).

$R^{2}=100 \times\left( \frac{\sum_{i=1}^{I} \sum_{j=1}^{J} \left( \hat{d}_{ij} \right)^{2}}{\sum_{i=1}^{I} \sum_{j=1}^{J} d_{\mathrm{ij}}^{2}} \right)$	(3)

$lof =100 \times\sqrt{\frac{\sum_{i=1}^{I} \sum_{j=1}^{J} \left( d_{\mathrm{ij}}- \hat{d}_{\mathrm{ij}} \right)^{2}}{\sum_{i=1}^{I} \sum_{j=1}^{J} d_{\mathrm{ij}}^{2}}}$	(4)

where d_ij ._ (i=1…I, j=1,…J), refer to the experimental data values of the analyzed data matrix and $\hat{d}_{\mathrm{ij}}$ refer to the corresponding MCR-ALS calculated data values. More details about MCR-ALS are given in previous works [8, 11, 21].

Table S1. Polyethylene standards and samples specifications, including their sample code, origin (manufacturer), and size range (µm).

| **Sample code** | **Origen** | **Size range (µm)** |
| --- | --- | --- |
| S1 | Cospheric | 45-53 |
| S2 | Cospheric | 106-125 |
| S3 | Cospheric | 300–355 |
| S4 | Cospheric | 500-600 |
| S5 | Cospheric | 850-1000 |
| K1 | Scientific Polymer Products (Sample Kit #205) | 14.8 |
| K2 | Scientific Polymer Products (Sample Kit #205) | 13.1 |
| K3 | Scientific Polymer Products (Sample Kit #205) | 11.7 |

Table S2. Instrumental parameters including source settings, tune conditions, and TIMS acquisition parameters.

| **SOURCE** | | | |
| --- | --- | --- | --- |
| **Source** | VIP-HESI-APCI | **Probe Gas Temp** | 250 °C |
| **End Plate Offset** | 500 V | **Probe Gas** | 3.0 L/min |
| **Corona** | 3500 nA | **Capillary** | 3500 V |
| **Nebulizer** | 3.0 Bar | **Dry Temp** | 250 °C |
| **Dry Gas** | 8.0 L/min | **Exhaust** | On |
|  |  |  |  |
| **TUNE-GENERAL** | | | |
| **Deflection 1 Delta** | 60.0 V | **High Sensitivity Detection** | Off |
| **Funnel 1RF** | 250.0 Vpp | **Funnel 2 RF** | 200.0 Vpp |
| **isCID Energy** | 0.0 eV | **Multipole RF** | 200.0 Vpp |
| **Ion Energy** | 5.0 eV | **Low Mass** | 60.00 m/z |
| **Collision Energy** | 10.0 eV | **Collision RF** | 450.0 Vpp |
| **Transfer Time** | 65.0 µs | **Pre Pulse Storage** | 3.0 µs |
|  |  | **Stepping** | Off |
| **TUNE-TIMS** | | | |
| **Dt1** | -20.0 V | **Dt2** | -120.0 V |
| **Dt3** | 50.0 V | **Dt4** | 50.0 V |
| **Dt5** | 0.0 V | **Dt6** | 20.0 V |
| **Funnel 1RF** | 300.0 Vpp | **Collision Cell In** | 140.0 V |

Table S3. CCS calibration details using Agilent tune mix reference ions, including experimental and reference CCS values, relative errors, and m/z values.

|  | **Experimental 1/K_0_** | **Error [%]** | **Reference 1/K_0_** |  | **Reference**  **m/z** | **Experimental m/z** | **Ionization** | **z** |
| --- | --- | --- | --- | --- | --- | --- | --- | --- |
|  | 0.5502 | -0.01 | 0.5446 |  | 188.0865 | 118.0865 | Positive | 1 |
|  | 0.7360 | -0.04 | 0.7363 |  | 322.0481 | 322.0471 | Positive | 1 |
|  | 0.9932 | 0.17 | 0.9915 |  | 622.0290 | 622.0279 | Positive | 1 |
|  | 1.1969 | 0.14 | 1.1986 |  | 922.0098 | 922.0075 | Positive | 1 |
|  |  |  |  | **Equation** | | | | |
|  |  |  |  | CCS = 189.74(1/K_0_) + 16.18 | | | | |
|  |  |  |  | **R^2^** | | | | |
|  |  |  |  | 0.999 | | | | |

Table S4. MS-DIAL parameters used for the confirmation of the compound’s identification.

| **MS-DIAL PARAMETERS** | |
| --- | --- |
| Ionization type | Soft ionization |
| Separation type | Chromatography + Ion mobility |
| Collision type | CID/HCD |
| Data type | Centroid data |
| Ion mode | Positive ion mode |
| Target omics | Metabolomics |
| Mass accuracy (tolerance) | 0.01 (MS1), 0.05 (MS2) Da |
| Minimum peak height | 500 amplitude |
| Mass slice width | 0.1 Da |
| Smoothing level | 3 scan |
| Minimum peak width | 5 scan |
| Retention time tolerance (alignment) | 0.1 min |
| Drift time tolerance (alignment) | 0.02 1/k0 |

Figure S1. Comparison between MSroi (left) and IMroi (right). Total ion Chromatograms (TIC, top) and corresponding raw data (bottom) for sample S1 (data example 1).

Figure S2. Comparison of LC-MS and IM-MS resolution Figure S2A shows that C4 (in LC-MS) is resolved into two distinct MCR components (C15 and C17 in IM-MS). Figure S2B shows how C7 in LC-MS is resolved into C5 and C19 in IM-MS. MCR resolved elution profiles are plotted on the left whereas MCR resolved spectra profiles are plotted on the right. This comparison illustrates how the incorporation of mobility profiles within the ROIMCR framework enables the separation of unresolved components in LC-MS data.

Figure S3. MS1 IM (A) and MS2 IM (B) data sets. Sequential (up), overlaid (middle) and Total Ion Current (bottom) plots are shown at each MS1 (left) and MS2 (right) levels for data example 2 (S1, S2, S3, S4, S5). These datasets illustrate the complexity of the IM-MS data structure addressed by the ROIMCR workflow.

Figure S4. MS1 IM (A) and MS2 IM (B) data sets. Sequential (up), overlaid (middle) and Total Ion Current (bottom) plots are shown at each MS1 (left) and MS2 (right) levels for data example 3 (Kit #205 samples K1, K2, and K3). These datasets illustrate the complexity of the IM-MS data structure addressed by the ROIMCR workflow.
